# Iraq War mortality estimates: A systematic review

**DOI:** 10.1186/1752-1505-2-1

**Published:** 2008-03-07

**Authors:** Christine Tapp, Frederick M Burkle, Kumanan Wilson, Tim Takaro, Gordon H Guyatt, Hani Amad, Edward J Mills

**Affiliations:** 1 Harvard Humanitarian Initiative, Harvard School of Public Health, Boston, USA; 2 Faculty of Health Sciences, Simon Fraser University, Burnaby, Canada; 3 Department of Medicine, University of Toronto, Toronto, Canada; 4 Department of Clinical Epidemiology & Biostatistics, McMaster University, Hamilton, Canada

## Abstract

**Background:**

In March 2003, the United States invaded Iraq. The subsequent number, rates, and causes of mortality in Iraq resulting from the war remain unclear, despite intense international attention. Understanding mortality estimates from modern warfare, where the majority of casualties are civilian, is of critical importance for public health and protection afforded under international humanitarian law. We aimed to review the studies, reports and counts on Iraqi deaths since the start of the war and assessed their methodological quality and results.

**Methods:**

We performed a systematic search of 15 electronic databases from inception to January 2008. In addition, we conducted a non-structured search of 3 other databases, reviewed study reference lists and contacted subject matter experts. We included studies that provided estimates of Iraqi deaths based on primary research over a reported period of time since the invasion. We excluded studies that summarized mortality estimates and combined non-fatal injuries and also studies of specific sub-populations, e.g. under-5 mortality. We calculated crude and cause-specific mortality rates attributable to violence and average deaths per day for each study, where not already provided.

**Results:**

Thirteen studies met the eligibility criteria. The studies used a wide range of methodologies, varying from sentinel-data collection to population-based surveys. Studies assessed as the highest quality, those using population-based methods, yielded the highest estimates. Average deaths per day ranged from 48 to 759. The cause-specific mortality rates attributable to violence ranged from 0.64 to 10.25 per 1,000 per year.

**Conclusion:**

Our review indicates that, despite varying estimates, the mortality burden of the war and its sequelae on Iraq is large. The use of established epidemiological methods is rare. This review illustrates the pressing need to promote sound epidemiologic approaches to determining mortality estimates and to establish guidelines for policy-makers, the media and the public on how to interpret these estimates.

## Background

On March 20, 2003, the United States invaded Iraq. In November 2002, four months prior to the invasion, MedAct published a report projecting that a US military offensive against Iraq could have enormous public health impacts in the short-, medium- and long-term [1]. The report estimated that the immediate, direct conflict-related casualties could, within only months of the start of a conventional war, range from 48,716 to 261,100 for Iraqi civilians, combatants and coalition forces combined, excluding the resultant indirect casualties and the possibility of a civil war.

In April 2003, the coalition forces removed Hussein's regime from power and US President Bush announced that major combat operations had ended [2]. As Hussein's Baath Party continued to disintegrate in the summer of 2003, the nature of the conflict began to shift. Although the U.S. declared major combat operations completed, insurgents continue to battle the coalition occupying forces using asymmetric warfare and guerilla tactics.

More than four years following the declaration that major combat operations had ended, violence in Iraq continues. Sectarian and insurgent attacks are responsible for the majority of current violence. Most attacks target civilians on the basis of their ethnicity or religion, or perceived affiliation with the new Iraqi government or occupying forces. The United Nations has stated that Iraq is on the brink of civil war [3].

Determining the total number of deaths resulting directly and indirectly from armed conflict is challenging. The war has caused the destruction of much of the health infrastructure and health information systems in Iraq, systems already weakened by UN sanctions in the decade prior [4]. As a result, attempts to determine the total number of Iraqi deaths have proved particularly problematic [5,6].

Iraqi government ministries, non-government organizations, academic institutions and media reporting agencies have attempted to estimate the total war-related deaths. The methods used to determine mortality have varied and, as a result, the mortality estimates are widely divergent. We critically examined the existing studies estimating the extent of Iraqi deaths and reviewed their methodological quality. We aimed to provide estimates of total mortality, average deaths per day, along with crude and cause-specific mortality rates attributable to violence. As a result, we chose a broad scope for the data abstraction.

## Methods

### Search strategy

We (CT, EJM, HA) conducted a systematic search of the following electronic databases from inception to January 2008: Allied and Complementary Medicine Database (AMED), Cumulative Index to Nursing & Allied Health Literature (CINAHL), Cochrane Library and Cochrane Central, Excerpta Medica (EMBASE), MedLine, Education Resources Information Center (ERIC), HealthStar, Agency for Healthcare Research and Quality – all through Ovid, Web of Science, and the United Nations websites (UNDP, UNHCR, UNICEF, WFP, WHO). We had no language restrictions and also specifically searched in Arabic. The key search term used was "Iraq". Where the volume of studies and reports produced was unmanageable, additional limits were set by adding the terms "casualties or deaths or mortality or civilian."

Additionally, we (CT, EJM, HA) conducted a non-structured review of ReliefWeb, Google Scholar and Google, recognizing the likelihood that investigators may have conducted research that they did not publish in health sciences information databases [7]. Detailed review of the references listed in key studies and reports provided an additional source of studies. Where unpublished reports or data were referenced, key individuals provided further information and access to these studies.

### Inclusion and Exclusion Criteria

We included studies and reports that assessed the total number of Iraqi deaths since March 20, 2003, the start of the war. Eligible studies incorporated Iraqi civilians and non-combatants in the total mortality estimates and were based on primary research done with the intent – either wholly or in part – of determining mortality estimates; studies that included combatants in addition to civilians and non-combatants were also included. We included unpublished primary research that was sufficiently reported via media sources and where key data could be extracted.

We excluded studies and reports that were limited to a specific sub-population (i.e. children under 5 years); however, studies were included if estimates on a specific sub-population were reported as a component of a larger total population number. Studies that reported only 'casualties' and did not separate out mortality estimates were excluded, as casualty reports may also include injuries. Reports of total mortality estimates which were included as part of a separate data source were not included, to reduce the possibility of duplicate reporting on the same data set. However, where methodologies differed and mortality estimates were not comparable, all studies were included.

### Data Abstraction

Using a priori defined study characteristics, we (CT, EJM) abstracted the following data from all studies: 1) date of data collection; 2) time period for reported deaths; 3) total number of deaths reported; 4) each of total combatant deaths, total civilian/non-combatant deaths; and, 5) cause-specific deaths attributable to violence, where available [Additional file 1]. In our review, 'mortality' refers to death from any cause, whereas cause-specific mortality confers the reported reasons for death, generally deaths due to violence. Additionally, data abstracted for the population-based surveys included methods of sample selection, sample size and pre-war data comparisons. For other studies, both published and unpublished, we also extracted information on study type and data source.

### Methodological Quality

There is a paucity of empirical evidence examining methodological quality in mortality estimates. We recently convened a methods workshop to determine recommended quality indicators in mortality surveys [8]. These methodological quality recommendations should only be applied to population-based studies and address issues of coverage, bias, completeness and accuracy. For the purpose of this manuscript, we extracted data on the following: a) whether the sample was sufficiently representative of the underlying population affected by the conflict; b) whether the population sampling avoided bias (i.e. was the random sampling systematic or simple); c) whether the response rate was reported; d) whether efforts were made to confirm deaths; and, e) whether households were revisited to confirm findings. Appendix 1 explains the rationale behind these quality indicators.

### Statistical Analysis

Given the heterogeneous methods and time periods applied to estimating mortality, we did not pool the included studies. Further, given the intense political interest in the mortality estimates, we provided details from each study. Where not already done by study authors, we (CT, EJM) calculated crude mortality rates (CMRs) and cause-specific mortality rates due to violence (VMRs) per 1,000 per year and average deaths per day based on the total mortality figures presented and time periods referenced in each study or report.

Mortality rates provide inferences on the average rate of death among a population over a specific time period. Where exact dates of birth and death events are not known, mortality rates are typically calculated as (*Total number of deaths during period/Population alive at the end of the period + half of deaths during period – half livebirths during period) × (rate multiplier/analysis period in time units of choice*). The rate multiplier (e.g. 1,000 or 10,000 people) and time unit (e.g. day, month, year) are chosen so as to express the rate in convenient units (in emergencies, typically per 10,000 people per day; in this paper, per 1,000 people per year). Where CMRs and VMRs were not provided by study authors, we calculated mortality rates for each study based on this formula, excluding livebirths as they are unknown. Our population estimates are based on the United Nations Development Program/Iraq Ministry of Planning and Development Cooperation population estimations for 2004. For all calculations we determined the total number of days in the study periods. Where the exact start and end date is either unknown or simply implied, we assumed either start, end or mid-month projections, indicated using ().

## Results

### Search Findings

Our systematic review and non-structured search together yielded a total of 19 relevant studies, surveys and reports at first pass. Of these, 6 studies were sourced through the systematic search and 13 through the non-structured searches. One study proved ineligible because the research objective was unrelated to determination of Iraqi deaths [9]. A multiple indicators cluster survey was also excluded because its findings were limited to infant and child mortality [10]. A report from the US Department of Defense [11] was removed as its estimates were sourced from other databases and no information was provided on the methodology; additionally, these figures are referenced in the dataset for the Brookings Institution, a study included in this review. In addition, a review done by the Los Angeles Times [12] was excluded to reduce the possibility of duplicate reporting, as it has been incorporated as a data source for one of the broader studies included in this review [13]. Information recently released by the American Civil Liberties Union was excluded because it was not a comprehensive mortality study and only provided information on fatalities that were reported to the US government for compensatory claims [14]. Lastly, a poll conducted by D3 Systems was excluded because it included any physical harms resulting from violence, not just deaths since the start of the war [15]. Our completed review includes a total of 13 studies.

### Study Characteristics

The 13 studies that we included are separated into two general categories: population-based studies and passive reporting. The passive data is further sub-divided according to whether they were published or unpublished (i.e. referenced only by secondary sources, usually the media). Tables 1, 2 and 3 [see Additional file 1] present study characteristics under each categorization. Each of the studies differed in terms of the mortality estimates, study populations, time period and research methodologies used, contributing to a broad range of mortality rates.

The time period for reported deaths ranged from 42 days, generally the duration of the 'war' as declared by the US government, to ongoing figures updated monthly or weekly. The study population ranged from only civilians/non-combatants to both combatants and civilians/non-combatants, included and excluded Kurdish regions, and reported deaths due to violence or from all causes.

### Study Quality

Only 5 studies clearly relied on a population-based approach; all were retrospective household surveys [16-20]. We found that all 5 studies provided appropriate details on representative sampling and used broad cluster sampling. All 5 studies reported on their methods of randomization in sampling and all used systematic random sampling to allocate clusters. All 5 studies reported on the response rates of households. Two of the studies attempted to ensure accuracy of the deaths by requesting death certificates [16,18]. Finally, 1 study reported revisiting households to confirm initial findings and found that there was a discrepancy between initial under-5 deaths reported and the larger number identified in the revisit [17,21].

### Individual Study Results

As there was a broad range of methodologies used, there are similarly a broad range of daily death rates, CMR and VMR totals. The average daily mortality rates ranged from 48 to 759, the Iraq Body Count (IBC) representing the lowest estimate and the Opinion Research Business (ORB) survey representing the highest. The cause-specific MR attributable to violence ranged from 0.64 to 10.25, with the IBC and ORB studies again representing each of the limits. Table 4 [see Additional file 2] presents detailed study outcomes.

### Individual Studies

Below, we summarize the key details from each study, separated according to population-based studies and passive reporting, and whether the data was published or unpublished.

### Population-based Studies

#### Roberts et al, 2004 (period of data collection March 20, 2003–September (15), 2004) [16]

This cluster random-sample survey of households throughout Iraq aimed to determine mortality of Iraqis before and after the 2003 invasion. Thirty-three cluster starting points were allocated among Governorates by probability proportional to size (PPS). In the sampling frame, adjacent Governorates (matched for pre-invasion violence and economic development) were paired to reduce travel time and the potential risk to study team members. Thirty households were then selected within each cluster. The starting household in the cluster was randomly selected using a GPS navigation system; research teams visited households adjacent to the starting households until a total of 30 were surveyed. Individuals were included in the survey if they had been residents of the household during the two preceding months. Research teams included medical doctors, both male and female, and all members spoke both English and Arabic. Each household was asked for information on births, deaths and visitors staying in the household for more than 2 months. This information was requested both for the period before (January 2002 to March 2003) and after (March 2003 to September 2004) the invasion. For reported deaths, interviewers requested copies of death certificates for two in each cluster, so as not to compromise researcher safety, as persistence may have been interpreted suspiciously. Death certificates were produced in 81% of the cases requested.

A total of 33 clusters, 988 households and 7,868 residents were chosen; five households refused to participate and in those clusters with proper absentee records, 872 households were visited (64 absent). A cluster located in Falluja yielded extremely high violent death rates, was considered an outlier and thus conservatively excluded in many of the analyses. The total mortality estimates include both civilians and combatants for all causes. The study estimates that there was an average of 98,000 excess deaths since the invasion (95% CI, 8,000–194,000). Approximately 51% were estimated to be attributable to violent causes, or 24% if Falluja is excluded. Based on these figures, there are an estimated 180 excess deaths per day (15–356). Study authors reported a crude mortality rate post-invasion of 12.3 (95% CI, 1.4–23.2) per 1,000 population per year over the entire study period, including Falluja, compared with a pre-invasion rate of 5.0 (95% CI, 3.7–6.3). Excluding Falluja, the total crude mortality rate post-invasion was 7.9 (95% CI, 5.6–10.2).

#### The Iraq Living Conditions Survey, 2004 (period of data collection March 20, 2004–May (31), 2004) [17]

The Iraq Ministry of Planning and Development Corporation, in partnership with the United Nations Development Program (UNDP), commissioned a multi-indicator household survey in 2004. The study was conducted by the NGO FAFO. Interviewers administered two questionnaires: 1) to recognized head of households on issues related to a variety of indicators, including housing and infrastructure, household economy, basic demography, and the education, health (including household deaths), and labour force characteristics of the household members; and, 2) to household women aged 15 to 45 on issues related to reproductive and child health. The survey was conducted in both Arabic and Kurdish. Although the survey was created in English, it was back-translated for verification. Authors estimated that the detailed survey took between 60 to 102 minutes per household. The gender mix and profession of those administering the questionnaire was not indicated.

The study was a multi-stage cluster sample survey. Cluster starting points were allocated among Governorates using a mixture of PPS and explicit stratification so as to provide various stratum-specific estimates (e.g. by Governorate and urban versus rural), based on population data from census or local statistics offices. Ten households within each cluster were then selected by segmentation (selection by PPS of a sufficiently small population unit, exhaustive listing of households in the unit, and systematic random sampling within this list).

The results of the surveys were sent to the Governorate office for registration and inspection, to Baghdad for coding, data entry and quality control, and then to FAFO's headquarters in Norway for further quality reviews. Where required, additional re-interviewing was conducted in the field.

A total of 21,668 households were surveyed from across Iraq, including all Kurdish areas. The total mortality estimates include both civilians and combatants. The ILCS estimates there were approximately 24,000 (95% CI, 18,000–29,000) war related deaths (presumably due to violence) since March 2003, as determined by the number of persons reported dead or missing in each of the households due to violent causes. Based on these figures, the violent death rate is 54 per day (41–66) and the violent mortality rate is 0.74 per 1,000 per year (95% CI, 0.55–0.89). The study investigators found discrepancies with a higher number of deaths reported when a sample of households was revisited [21].

#### Burnham et al, 2006 (period of data collection March 20, 2003–July (15), 2006) [18,22]

This study was intended to update the mortality figures reported by a previous study published in 2004 [16]. For this study, researchers conducted a cluster random-sample survey of households throughout Iraq. Fifty clusters of 40 households, estimating 8 members per household, were allocated among Iraq's Governorates using PPS. A further stage of PPS sampling allocated clusters to administrative units within each Governorate. Within each administrative unit, one main street and one residential street crossing this main street were randomly selected, and the starting household for the cluster selected randomly among all households along this residential street. Researcher teams then visited adjacent households until 40 households were surveyed. Individuals were included in the survey if they had been residents of the household during the three preceding months. Research teams were comprised of trained medical doctors, both male and female, who spoke both English and Arabic. Each household supplied information concerning births, deaths and in/out migration before and after the invasion. Wherever possible and where interviewer safety was not deemed to be compromised, the cause of death and a copy of the death certificate were requested for verification.

A total of 47 clusters, 1,849 households and 12,801 members were included in the final data analysis; 3 clusters were not completed due to miscommunication and other security issues, and were excluded from the final sample. Results were based on comparing the death rates in all households surveyed for the period before invasion (January 2002 to March 2003) and in the period after (March 2003 to July 2006) to determine the total excess deaths due to the war, meaning those deaths above the pre-invasion rates. Mortality estimates include both civilians and combatants for all causes. The study estimates that there were 654,965 excess deaths since the invasion (95% CI, 392,979 to 942,636). Of these, 601,027 (95% CI, 426,369 to 793,663) were attributable to violent causes. Based on these figures, there are an estimated 540 deaths per day (95% CI, 324 – 777). Study authors reported a crude mortality rate of 5.5 (95% CI, 4.3–7.1) in the period pre-invasion, 13.2 (95% CI, 10.9–16.1) per 1,000 population per year over the entire study period, and a rate of 19.8 (95% CI, 14.6–26.7) for the last year from June 2005 to June 2006. The overall cause-specific mortality rate due to violence is 7.2 per 1,000 per year.

#### Opinion Research Business, 2007 (period of data collection March 20, 2003–August (15), 2007) [19]

The British polling agency Opinion Research Business (ORB), in partnership with their Iraqi fieldwork agency, has been tracking opinion in Iraq since 2005 and conducted a poll of the population in August 2007 to determine the impact of the ongoing conflict.

ORB used a multi-stage random probability sample in 15 of the 18 governorates to derive a representative sample of 1,720 households. Karbala and Al Anbar governorates were excluded for security reasons and Irbil refused to grant a permit to conduct the survey. 1,499 in the sample agreed to respond to the question. The poll asked an adult over the age of 18 in each household: "How many members of your household, if any, have died as a result of the conflict in Iraq since 2003 (i.e. as a result of violence rather than a natural death such as old age)? Please note that I mean those who were actually living under your roof." The survey was conducted through face-to-face interviews. No further information regarding the study methods was provided.

Respondents replied by stating the number of individuals in the household that have died due to violence since March 2003: 78% stated none; 16% stated one; 5% stated two; 1% stated three; and, 0.002% stated four or more. ORB based their total mortality calculations on the 2005 census information, which assumes that there are a total of 4,050,597 households throughout Iraq. They estimate that 1,220,580 [margin of error +/- 2.5% equals a range of 733,158–1,446,063] deaths due to violence have occurred since March 2003. Based on these estimates, there are 759 deaths per day (range 456–899) and the violent mortality rate is 10.25 per 1,000 per year (range 6.15–12.16).

#### Iraq Family Health Survey, 2008 (period of data collection March 20, 2003–June (15), 2006) [20]

The Iraq Family Health Survey was conducted in 2006 and 2007 by federal and regional ministries in Iraq, and the Central Organization for Statistics and Information Technology (COSIT), with technical support provided by the World Health Organization. It was a cross-sectional, nationally representative household survey that estimated morbidity and mortality rates from January 2002 through June 2006, for both pre- and post-invasion periods.

The study design was a two-stage stratified sample survey of households. Excluding Baghdad, each of 17 of the 18 governorates in Iraq were divided into 3 sampling domains; Baghdad was divided into 5 sampling domains, for a total of 56 sampling domains in the survey. Each of the 56 domains was further stratified into 18 clusters and 10 households were selected from each cluster using a linear random systematic sampling. The total target sample was 10,080 households. However, cluster sizes in the Baghdad-Karkh, Anbar and Nineveh domains were increased to account for the liklihood that some clusters would be inaccessible due to insecurity. Following this increase, a total of 1,086 clusters and 10,860 households were selected to be surveyed.

115 clusters in Anbar, Baghdad, Wasit and Nineveh were not surveyed due to insecurity. Mortality estimates for these areas were imputed using data from the Iraq Body Count to determine the ratio of death rates relative to other comparable high mortality provinces. Due to the exclusion of these areas, 9,710 households were visited and 9,345 interviews were conducted for a total response rate of 96.2%. The interview teams were comprised of both men and women and some of the female interviewers were doctors or other health professionals.

Three questionnaires, translated into both Arabic and Kurdish, were administered to three different members of each household regarding mental health, reproductive health, and other general questions. Interviewers asked for information on each household death that occurred during the study period, including sex, age, time and place of death, as well as the primary cause, as reported by the respondent. Questionnaires and forms in each completed cluster in the South/Centre of Iraq were sent to the Federal Ministry of Health in Iraq for verification; incorrect or incomplete forms were sent back to the respective governorates. Data was double entered and verified. Death certificates were not requested for verification. Design weights were calculated to adjust for different household sampling selection probabilities as well as non-responses.

Crude and cause-specific mortality rates were calculated by comparing the exposure times (to the nearest month) to the risk of death for the living and deceased in each household between for the pre-invasion period (January 2002 to February 2003) and post-invasion periods (March 2003 to June 2006). Estimates for violent deaths post-invasion accounted for survey sampling errors, including adjusting for the missing clusters, underreporting, and projected population figures due to migration.

There were a total of 61,636 people living in the households that were sampled. After adjusting for the rates in excluded clusters, the crude death rate prior to the invasion for all of Iraq was calculated to be 3.17 [95% CI, 2.70–3.75] per 1,000 person years; the rate post-invasion was 6.01 [95% CI, 5.49–6.60] per 1,000 person years. The cause-specific death rate due to violence was 0.10 [95% CI, 0.04–0.32] and 1.09 [95% CI, 0.81–1.50] per 1,000 person years, respectively; however, after accounting for sampling errors and adjusting for missing clusters, the rate was projected to be 1.67 [95% CI, 1.24–2.30]. Authors estimate that the number of deaths due to violence from March 2003 through June 2006 is 151,000 [95% CI, 104,000 to 223,000]. Based on this, the number of violence-related deaths per day is approximately 126 [95% CI, 87–186].

### Passive reporting

#### Published Studies

##### Conetta C, Project on Defense Alternatives Research Methods (PDAR), 2003 (period of data collection March 19, 2003–May 1, 2003) [13]

The PDAR conducted an analysis and synthesis of data reported on Iraqi deaths, including journalistic surveys of casualty incidents, hospitals, burial sites and graveyards and battlefield eyewitness accounts from embedded journalists and military personnel. Totals are based on data extrapolation for all of Iraq, based on the database of compiled reports and incidents.

Civilian casualty data was generally compiled from media surveys and reports, including those by the *Associated Press, Knight-Ridder *and the *Los Angeles Times *and supplemented by NGO studies, such as the Campaign for Innocent Victims in Conflict (CIVIC), as well as other media reports of casualty incidents based on eyewitness accounts from hospital personnel, aid workers, and the families of the dead. Mortality estimates for combatants are derived largely from embedded journalists and interviews with military personnel on both sides and some estimates based on artillery power projections. PDAR has reported total figures for all deaths and those separately for both non-combatant and combatants. The authors do not provide a specific time period; all estimates are for deaths that occurred, generally, "during the war", presumably the period from invasion to the official declaration by President Bush that combat had ceased (May 2003).

The PDAR estimates that a total of 12,950 Iraqis were killed in the war (+/- 2,150 a range of 15,100–10,800). Of these, 9,200 (+/- 1,600 a range of 10,800–7,600) were combatants and 3,750 (+/- 500 a range of 4,250–3,250) were non-combatants. However, PDAR has recognized that in some cases there may be overlap and an inability to distinguish precisely between combatant and non-combatant deaths. Based on these figures, the total daily death rate would be 308 per day (range, 287–360) with a crude mortality of 4.15 per 1,000 per year (range, 3.46–4.84); separated out, the rate for combatants would be 219 deaths per day (range, 181–257) with a crude mortality rate of 2.95 (range, 2.43–3.46) and non-combatants 89 deaths per day (range, 77–101) and a cause-specific mortality rate due to violence of 1.20 (range, 1.04–1.36) per 1,000 per year.

##### United Nations Assistance Mission for Iraq (UNAMI), December 2006 (period of data collection May 1, 2006–December 31, 2006) [23]

UNAMI is a centralised information sharing group which supports the UN's continued involvement in Iraq following the conclusion of the Oil for Food program.

The UNAMI Human Rights Office produces monthly reports identifying a series of current and ongoing rights violations as well as recommendations intended to mitigate the impacts. Included in these reports are total per-period deaths. Mortality figures are determined by combining the deaths reported by the Ministry of Health (MOH) and the Medico-Legal Institute of Baghdad (M-LIB). The MOH derives their reports from mortality figures received by all hospitals in Baghdad and other Governorates, with the exception of Kurdistan. The M-LIB totals are based on bodies brought to the morgue. The reported totals are for civilian deaths only and are almost entirely due to violence (with the exception of approximately 5–6% of the M-LIB figures).

UNAMI reported 25,847 total deaths in reports during the period of May to December, 2006. Although reports are also available from July 2005 to April 2006, they have been excluded for purposes of this review due to gaps in data and inconsistently reported methodology. In addition, the quarterly report for January to March 31, 2007, states that the Iraqi Government declined to provide access to the Ministry of Health's overall mortality estimates for this period. Based on the total deaths reported between May and December 2006, the number of deaths per day is approximately 106 and the cause-specific mortality rate attributable to violence is 1.42 per 1,000 per year.

##### The Brookings Institution, January 2008 (period of data collection May, 2003–December (15), 2007 and January 16, 2008) [24]

The Brookings Institution (BI) is an independent research organization that analyzes US national public policy issues. The 'Iraq Index' report is prefaced by stating that, generally, data is derived from the US government, NGOs and media reports, with only a small amount of information coming from Iraqi sources. Some of the data also includes further models by the BI.

The military and police mortality data referenced the Iraq Minister of the Interior, military personnel and media reports as information sources. The total number of Iraqi military and police deaths reported from June 2003 to January 16, 2008 is 7,792.

Data for civilian deaths was based on figures provided by the Iraq Body Count for the period from May 2003 to December 2005. This data was increased by a factor of 1.75, to account for the difference in casualty estimates noted between the IBC and Iraqi Ministry of the Interior for this period. The Brookings Institution conducted a separate study of the crime rate and estimated that approximately 23,000 murders had occurred throughout Iraq; the IBC and crime data were combined for a monthly estimate from May 2003 to December 2005 (44, 265). From the period of January 2006 to December 2006 mortality figures from UNAMI were totaled (34,452). Estimates for the period from January to December 2007 were based on U.S. State Department Weekly Status Report, September 12, 2007 and press briefings (18,300). The entire period civilian mortality estimate is based on a sum of these numbers and totals approximately 97,017.

Based on this information, the military and police daily mortality rate was approximately 5 deaths per day with a cause-specific MR due to violence of 0.06; the death rate for civilians was 57 deaths per day and cause-specific MR due to violence of 0.77 per 1,000 per year.

##### Iraq Body Count, January 2008 (period of data collection March 20, 2003–January 17, 2008) [25]

The Iraq Body Count (IBC) is the source most often referenced by coalition-forces politicians for Iraqi mortality estimates [26]. The IBC maintains a publicly accessible database of civilian deaths due to violence and increasing lawlessness resulting from the war, based on online surveys and compilation of media-reported deaths. Totals are based on mortality figures reported by online English language media agencies, designated from a list that meets IBC determined baseline standards and which are validated by at least two independently reported sources. Where discordant figures are reported, a range of deaths is provided by IBC. Although all figures are checked by two IBC staff and the original compiler of the data, the accuracy ultimately relies both on deaths actually reported by the media and on the rigor of the reporting agency. As such, IBC has acknowledged that total figures reported are likely underestimated [27].

As of mid-January 2008, the IBC reported an average of 84,333 Iraqi civilian deaths (range 80,621 to 88,044). Based on this figure for approximately a 1,764 day period, the average death rate would be estimated at 48 deaths per day (range 46 to 50) and the cause-specific MR due to violence is 0.64 per 1,000 per year (range 0.61 to 0.67).

##### Just Foreign Policy, January 2008 (period of data collection March 20, 2003–January 17, 2008) [28]

Just Foreign Policy (JFP) is an independent organization that promotes reformation of U.S. foreign policy. The group keeps an ongoing and continually updated record of Iraqi civilian deaths due to the U.S. invasion, intended to be a "daily rough estimate" and not a scientific study.

JFP estimates are based on a combination of the results of the Burnham et al. study and the Iraq Body Count. Mortality estimates are calculated according to the following formula: [Burnham et. al estimate as of July 2006] × [(Current IBC Deaths)/(IBC Deaths as of July 1, 2006)] As of mid-January, JFP projected that there were 1,168,058. Based on this, there are approximately 662 deaths per day and the mortality rate is 8.95 per 1,000 per year.

#### Unpublished Studies

##### The People's Kifah, 2003 (period of data collection March 20, 2003–October (15), 2003) [29,30]

The People's Kifah, an Iraqi political organization, is reported to have conducted a population-based study in September and October 2003. Volunteers went into villages, towns and cities across Iraq in 14 Governorates, excluding Kurdish areas, to collect death statistics and information from individuals and hospitals. The survey also included interviews with grave-diggers and other eyewitness accounts. As the actual survey is unpublished, figures are derived from media reports on communications and statements sourced from the organization's spokesman, Dr. Muhammad al-Obaidi [30]. Unfortunately, the survey is reported to have discontinued due to the kidnapping of one of the workers.

The People's Kifah reported 37,137 civilian deaths due to violence during the seven months from March to October 2003. Therefore, the daily death rate is 177 and the cause-specific MR due to violence is 2.39 per 1,000 per year.

##### Iraqiyun, 2005 (period of data collection March 20, 2003–July 11, 2005) [31]

Iraqiyun is a humanitarian organization lead by Dr. Hatim al-Alwani. The actual study produced by the organization is unpublished, therefore total deaths are based on press reporting of statements made by al-Alwani which have implied that the study may have been a population-based study. Iraqiyun figures are based on reports from hospitals across the country and information obtained from relatives and families of the deceased.

On July 12, 2005, the organization reported that 128,000 Iraqis had died since the start of the war. Based on this figure, there are 152 deaths per day and a cause-specific mortality rate due to violence of 2.03 per 1,000 per year.

##### Iraq Ministry of Health, 2006 (period of data collection March (20), 2003–November (30), 2006) [32]

In November 2006, Iraqi Health Minister Ali al-Shamari estimated that approximately 100,000–150,000 Iraqi's had been violently killed since the start of the war. Ministry staff subsequently confirmed these figures.

Ministry totals are based on daily death counts reported at hospitals across the country and morgues in the Baghdad area, although no actual reports are published or publicly available. The Ministry began collecting mortality data in 2004, so the total estimate is based on currently reported daily death rates that have been extrapolated back to March 2003. The figures include all deaths (combat/non-combat) by violent causes. The reported daily death rate ranges from 75 to 100 per day, with a total of 100,000 to 150,000 estimated total deaths since the start of the war and a cause-specific MR due to violence of 1.01 to 1.34 per 1,000 per year.

## Discussion

Estimating mortality during conflicts, such as the Iraq war, presents important obstacles. Appropriate methods exist, such as population-based methods of retrospective surveys and community-based prospective surveillance, and are widely available on the internet and medical literature [27,33]. However, despite the availability of appropriate and accepted methodologies, studies we reviewed here used diverse methodologies and were of widely divergent quality – some with major weaknesses, and, presumably therefore resulting in differing estimates of mortality. Nevertheless, the media frequently reports on these estimates and they can have an important influence on the political process and subsequent foreign policy decisions [26]. Given the importance of these reports, it is concerning that there is such a wide variance in their methods and estimates.

We have systematically reviewed the literature to identify all of the estimates of mortality and describe their methodology to bring more transparency to this critical area of epidemiology. Our review identified a total of 10 published studies that included 5 population-based studies [16-20] and 5 passive reporting studies [13,23-25,28]. Only 3 studies have been published in peer-reviewed sources [16,18,20], although non-journal based publications may also hold to academic standards. We also identified 3 additional studies based on secondary reports with no identifiable source document [29,31,32]. The studies provided mortality estimates varying from 48 deaths per day to 759 deaths per day. Although daily death rates and violent mortality rates have been calculated for each study, these figures should serve only as a general framework because they obscure the methodological differences in each study and surges of violence that may occur at specific times. For example, some studies limited mortality totals to civilians/non-combatants, while others included combatants; some studies included only violent deaths, others included deaths for all causes. Some also excluded Kurdish areas of Iraq, while others did not. In addition, the different methods for gathering data each inherently have strengths and limitations that affect the range, whether higher or lower, of mortality estimates produced (See Appendix 2). There are, as yet, no tools to critically appraise the quality of evidence from armed conflict studies and it is clear that there is a pressing need for the development of appraisal checklists, and further consideration of systematic reviews/meta-analyses of humanitarian emergency data [34].

The two broad classes of data collection methods, population-based and passive reporting, partly explain the variance in the estimates (See Appendix 2) [27]. The population-based methods are well established and a generally accepted method within the fields of epidemiology [27,33]. Studies using a population-based method are more sensitive for estimating mortality, by identifying non-reported deaths.

All of the 5 included studies [16-20] that used population-based methods also used established methods to reduce investigator-driven bias, including random sampling, *a priori *sample size estimation, *a priori *specification of locations and PPS; four reported on sampling imprecision through presentation of findings with confidence intervals [16-18,20]; and, three attempted to acquire accuracy of deaths [16-18]. Two of the population-based studies requested death certificates to verify causes of death and were successful in obtaining them in the vast majority of reported deaths [16,18]. While such methods may yield accurate estimates of national rates by sampling only a small proportion of the population, this accuracy is critically dependent on the representativeness of the sample. However, such methodologies are less specific in identifying causes of death and may be susceptible to reporting and sampling biases. This is most appropriately addressed through the requests for death certificates. Compilation from primary sources or passive reporting methods, that rely upon media and/or official sources for mortality information are likely to be more specific, however, would be expected to considerably underestimate true mortality by not capturing unreported deaths and indirect deaths, from non-violent effects of war, for example, that are not often attributed to the ongoing conflict [35].

Of the population-based studies, the Roberts and Burnham studies provided the most rigorous methodology as their primary outcome was mortality [16,18]. Their methodology is similar to the consensus methods of the SMART initiative, a series of methodological recommendations for conducting research in humanitarian emergencies [33]. Another population-based study, the Iraq Living Conditions Survey, reported lower death estimates that we assume is due to the survey being conducted barely a year into the conflict, a higher baseline mortality expectation, and differing responses to mortality when houses were revisited [21]. However, not surprisingly their studies have been roundly criticized given the political consequences of their findings and the inherent security and political problems of conducting this type of research [36,37]. Some of these criticisms refer to the type of sampling, duration of interviews, the potential for reporting bias, the reliability of its pre-war estimates, and a lack of reproducibility. The study authors have acknowledged their study limitations and responded to these criticisms in detail elsewhere [38]. They now also provide their data for reanalysis to qualified groups for further review, if requested.

Recently, a study published in the New England Journal of Medicine estimates that there were approximately 151,000 violence-related deaths from March 2003 to June 2006. The authors estimate that the completeness in reporting of deaths was 62% and that the underreporting for violent deaths may be as much as 50%. In addition, the authors did not indicate whether verification was sought by requesting death certificates and there may be a resistance of some household members to disclose cause of death. In a previous study conducted by members of the research group, household re-visits determined that there was under-reporting in the under-5 mortality rate.

Of the passive surveillance studies the IBC study was, until recently, the most frequently cited by media sources and coalition force politicians [26]. The IBC was largely established as an activist response to US refusals to conduct mortality counts. This account, however, is problematic as it relies solely on news reports that would likely considerably underestimate the total mortality. This method does not count indirect deaths, such as increased chronic illness due to the war, or deaths that are not publicly reported. More recently, the media has relied on Iraq Ministry of Health reports that estimate 75–100 deaths per day, and a cause-specific mortality rate due to violence of 1.01 to 1.34 per 1,000 per year [32]. This report, based on total daily deaths/body counts from hospitals across Iraq and the Baghdad morgue, is likely to provide a more accurate estimate than the IBC, but would similarly not identify indirect deaths or deaths not reported to the health facility [36]. There is the potential that this report may overestimate the early war death toll because it extrapolates current daily death rates to produce a death total and mortality rates have steadily increased during the most recent months. However, like other passive reporting studies it would again underestimate the total mortality by not capturing unreported and indirect deaths. The utility of such collection data serves strictly as a 'sentinel case' alert that should prompt further population-based cluster sampling before such findings are widely disseminated or quoted as fact.

There have been ongoing discussions and disagreements regarding the most reliable figures derived from these studies, including extensive deconstruction of the methodological merits of each [39,40]. It is well known that collecting mortality data during times of protracted violence faces inherent challenges of investigator, participant, and data security. The People's Kifah study exemplifies the dangers involved in data collection related to conflict settings, as they reported that the study was terminated due to the kidnapping of a data collector.

There are several strengths and limitations to consider in interpreting this review. Strengths include the extensive searching and identification of studies that met our inclusion criteria. Other reviews and websites summarizing Iraq war deaths have also reported on estimates produced by the NGO Coordinating Committee [27]. This estimate has, however, never been made public and sources citing it have had difficulty confirming its existence. We were successful in communicating with the Coordinating Committee and they have verified that this data does not exist (Cedric Turlan, personal communication, Dec 2006). We attempted to communicate with the authors of all published population-based studies.

There are also several limitations to consider in this review. As this topic is highly politicized, it is possible that mortality studies exist but have not been made publicly available. Indeed, as we found with the Iraqiyun and People's Kifah studies – both whose study results have been reported in the media but where the primary research is unpublished – there is reason to believe that further mortality estimates may exist. Ascertaining the reason for death is a somewhat specious endeavourer as participant recall and interpretation inevitably influences their reported reasons for death. While we would expect violent mortality to be more clearly understood than infectious diseases, for example, we recognize that the same victims affected by violent injuries may also die from diseases that are pre-existing or acquired.

Our denominator for calculating mortality rates is based on the United Nations Development Program/Iraq Ministry of Planning and Development Cooperation estimations for 2004. It is possible that this estimate is slightly misleading as we would expect some further population displacement since 2004, through either immigration or internal displacement. Finally, we aimed to review the methodological quality of the included studies. Although these quality indicators are based on consensus, there is a general lack of empirical data to guide recommendations of minimum reporting of methods and results [41]. It is possible that other methodological items may be more important and we plan to further address these issues in upcoming work.

It has yet to be determined how long the sectarian violence in Iraq will continue and what the impact will be in terms of total mortality. The results of this systematic review demonstrate that there are immensely different mortality estimates. However, the volume of studies produced also indicates that the international community is attentively watching events unfolding in Iraq. The studies and reports show that, despite varying estimates, large numbers citizens in Iraq are still dying as a result of the war. With the growing body of data, politicians and policy makers are remiss to ignore the evidence and make policy decisions that do not consider the health, safety, security and rights of the citizens they claim to protect.

## Conclusion

Despite the variance in estimates it is clear that the Iraq war has exacted a large human cost. However, the variance in estimates and their influence in policy and public opinion underlines the importance for critical appraisal tools and guidelines to be established to ascertain the strength of inferences provided through each methodology. Efforts should also be made to ensure studies are being conducted in other conflict zones to provide real time feedback on the impact of current policies [42]. A recommendation made by UNHCR in 2001, that a regulatory committee exist to interpret complex emergency data and humanitarian data, is clearly desperately needed, to avoid unreasonable political or humanitarian responses [42]. Finally, the media should be educated on the different types of studies so as to explain to the public why mortality estimates vary and be able to comment accurately on study estimates, strengths and weaknesses.

## Competing interests

Edward Mills has previously received funding from the Canadian Government to conduct methodological research on mortality estimates. Frederick Burkle was the interim Minister of Health for Iraq during the crisis period in 2003. No funds were provided for this study.

## Appendix 1: Quality considerations for retrospective surveys

Coverage: Was the population sampled sufficiently representative of the underlying population affected by the conflict?

This question determines whether the households or settings visited appropriately represent populations affected by the conflict.

Bias: Was the population sampled to avoid bias?

As some populations will be more, or less, affected by the conflict, study investigators should make efforts to reduce bias in sampling the population. The most common manner to reduce bias is random selection of participating households.

COMPLETENESS: Is the response rate reported?

The response rate provides inferences on how likely the population sampled may represent the overall population. Is there reason to believe that those that did not respond are systematically different than those that did respond?

ACCURACY: Were efforts made to confirm deaths?

Did the study investigators aim to confirm deaths through corresponding evidence, eg. Death certificates.

Was a sample of households revisited to confirm findings?

Did the study investigators revisit a random sample of study households or settings to re-inquire about deaths and did they find the same results?

## Appendix 2: Common features of mortality study methodologies

### Population-based mortality studies

Population-based studies, when conducted well, provide strong inferences regarding the impact of a long-term conflict upon a large population. Time periods over which events take place are of prime importance in population-based case finding. Population-based case finding based on survey estimation consists of drawing a representative sample of households, and, based on a questionnaire, eliciting information about the demographic evolution of household members over a specified retrospective period; mortality rates are estimated based on deaths reported in the sample over the recall period, and total person-time of exposure sampled (roughly equivalent to the number of individuals included in the sample times the recall period); confidence intervals are presented alongside point estimates. Population-based case finding can also be performed through prospective surveillance, whereby investigators collect community-based data on deaths on an ongoing basis. Accuracy of estimates from surveys depend on the representativeness of sampling and accuracy of reports from respondents. Efforts are required to ensure the specificity of causes of death and the reliability of death occurring with the specific time period. As with all studies, use of a control-group strengthens the inferences regarding how likely the outcomes are due to chance alone. One may wish to compare mortality rates among the target population with mortality rates from a nearby population, or from a geographically distinct population. Difficulties arise in choosing a control group, as populations affected by conflict may be systematically different from a population that is not affected by conflict, for reasons such as ethnicity, political representation and geographic location, among others. For that reason, choosing a control group risks having a spuriously unrepresentative control and other options must be sought. One option is to consider using the same population (as own control) as a pre and post exposure population. Although self-controls are a weak design in therapeutic research, when used with caution, they can present compelling inferences in epidemiological surveys. While the methodology for evaluating mortality are still in development, some recent examples of retrospective surveys exist for major conflicts including Iraq, Darfur and DRC [16,42,43].

### Passive reporting mortality studies

These studies involve monitoring trends in real-time and requires continuous surveillance of deaths as they occur in a region and is ideal for prompting a quick response. This is generally considered to be a passive system of reporting deaths as the death or body must reach the point of measurement, eg. a hospital or morgue. Team members, usually local community members, are each assigned an area in which they are to record deaths. They may seek information from facilities regularly, such as weekly or even daily, or they may wait for deaths to be reported to them. Mortality rates are calculated by dividing the total number of deaths in a period divided by the total population at risk in the region and the period of analysis.

Passive reporting studies should be interpreted as 'sentinel data' that provides strong inferences about the likelihood of atrocities or violence. This data collection should then be confirmed with population-based case finding to determine the magnitude of the issue. Passive reporting will generally under-report death rates, particularly when access is restricted or events are intentionally hidden. Alternatively, they could also over-report events, if there is a political reason to make exaggerated claims. Passive reporting systems have, in the past, under-reported deaths by as much as 10-fold, when compared to retrospective population-based surveys [44]. If passive reports are used, then it is necessary to use independent methods to assess the reliability of reporting. These may include on-going active surveillance, auditing by independent evaluation groups, or triangulation with other geographic regions also affected by violence.

Population-based and passive methods often go together and help generate a more complete picture of mortality trends over time. In this context, surveys are defined as assessments of specific populations at specific time points, whereas surveillance refers to ongoing assessment and interpretation of health data to inform policy [45]. Both types of methods have their advantages and disadvantages and so deciding which method to employ depends on several factors including length of recall period, whether the population size is known, and the types of resources available.

## Authors' contributions

FB, CT, KW, TT, GHG, HA, and EJM contributed to manuscript development and to the development of the quality indicators. CT and EJM conducted the database search and data abstraction for all studies. HA conducted the database search in Arabic. All authors read and approved the final manuscript.

## Supplementary Material

Additional file 1 Table 1: Study Characteristics, Population-based studies; Table 2: Study Characteristics, Passive reporting – published studies; Table 3: Study Characteristics, Passive reporting – unpublished studies. These three charts outline the study characteristics abstracted from each of the included studies, counts, and reports. Click here for file

Additional file 2 Table 4: Study Results. This chart presents the detailed study outcomes, including mortality totals and rates. Click here for file
