# Supplementary material for: Iraq War mortality estimates: A systematic review
Source: Confl Health. 2008 Mar 7;2:1. doi: 10.1186/1752-1505-2-1 (PMC2322964; doi:10.1186/1752-1505-2-1)
Supplement: Additional file 1 — Table 1: Study Characteristics, Population-based studies; Table 2: Study Characteristics, Passive reporting – published studies; Table 3: Study Characteristics, Passive reporting – unpublished studies. These three charts outline the study characteristics abstracted from each of the included studies, counts, and reports. [file 1752-1505-2-1-S1.doc]

Table 1: Study Characteristics

*Population-based studies*

| **Author(s)** | **Study Type** | **Sample Selection** | **Sample Size** | **Survey Methods** | **Included/Excluded** | **Validation Tools** | **Pre-War Data Comparison** |
| --- | --- | --- | --- | --- | --- | --- | --- |
| Roberts  2004 | Survey-based; cluster sample survey (before and after comparisons), no stratified sampling. | Sample households and start household randomly selected by by cluster sampling. Clusters assigned to paired Governorates (to minimize travel time) by probability proportional to size.  Start household randomly selected by GPS navigation system and following households selected using a rule of proximity. | 33 cluster areas with 30 households and estimated 7 members each were randomly selected from paired Governorates.  33 cluster areas, 983 households and 7868 members were surveyed; 5 households refused to participate. | Team of bilingual researchers, including both males and females, conducted household interviews on deaths, births and household visitors. | All deaths included.  Falluja excluded as an outlier in some data calculations. | Death certificates sought for 2 deaths in each cluster; produced in 81% of cases where requested. | Mortality rates were calculated for period pre-invasion and post-invasion based on the survey results (to Sept. 2004). The CMR for the pre-war period was 5.0.  The excess mortality in post-invasion period was calculated based on modelling relative risk of death pre- and post-war in each cluster. |
| Iraq Living Conditions Survey  2004 | Survey-based; multi-stage cluster sample survey. | Cluster sample, with household selection at the last stage based on sub-segmentation, mapping and systematic sampling of chosen segment (community within which cluster was to be drawn). Explicit stratification with post-sampling weighting. | 1,100 households selected from each Governorate; Baghdad included 3,300 households.  A total of 22,000 households were selected; 21,668 were actually interviewed. | Survey was conducted in both Arabic and Kurdish.  Two questionnaires were administered: 1) to the recognized head of the household related multiple indicators and 2) to women of the household, aged 15-45 related to reproductive and children’s health. | All deaths included.  Includes Kurdish areas. | Information sent to Governorate office for registration and inspection.  Quality control done during coding and data entry in Baghdad.  Where/if needed, re-interviewing was done; in some cases, such as for <5 mortality, a 50% under-reporting was noted.  Completed files sent to headquarters in Norway for quality checks. | N/A |
| Burnham  2006 | Survey-based; multi-stage cluster sample survey (before and after comparisons), no stratified sampling. | Cluster sampling, start household selected by random selection of main streets then residential streets within community and random selection (by number assignment) of one household along the chosen street; remaining households selected by a rule of proximity (each adjacent one). | 50 clusters with 40 households randomly selected by Governorate, population proportional to size.  47 clusters of 40 households, 1849 households and 12,801 members included in final data analysis; 15 households refused to participate. 3 clusters were misattributed due to miscommunication and security issues and were excluded from the final sample. | Team of bilingual researchers, including both males and females, conducted household interviews on deaths, births and migration in/out. | All deaths included. | Deaths confirmed reported by death certificate. | Mortality rates were calculated for the period pre-invasion and at approximately one year intervals in the years following, based on the survey results. The CMR for the pre-war period was 5.5.  The excess mortality in post-invasion period was calculated based on modelling relative risk of death pre- and post-war in each cluster. |
| Opinion Research Business  2007 | Survey-based; multi-stage random probability sample in 15 of 18 governorates. | Representative sample size selected through random probability sampling from 2005 census information that indicates there are a total of 4,050,597 households across Iraq. | 1,720 households selected; 1,499 agreed to participate. | Face-to-face interviews conducted; interviewers asked: “*How many members of your household, if any, have died as a result of the conflict in Iraq since 2003 (i.e. as a result of violence rather than a natural death such as old age)? Please note that I mean those who were actually living under your roof.*” | Karbala and Al Anbar excluded due to insecurity; Irbil did not grant permit. | Information not provided. | N/A |
| Iraq Family Health Survey  2008 | Survey-based; cross-sectional, two-stage stratified, nationally representative sample household survey (before and after comparisons) | Governorates divided into 3 sampling domains each (Baghdad divided into 5) and each sampling domains further stratified into clusters; households were selected from each cluster using linear random systematic sampling. | There were a total of 56 sampling domains that were stratified into 18 clusters and 10 households selected from each cluster; target sample was 10,080 households, but additional clusters were added to Baghdad-Karkh, Anbar and Nineveh domains to account for likely inaccessibility due to insecurity.  Total of 1086 clusters and 10,860 households selected; 9710 households were visited and 9,345 interviews conducted. | Survey was translated in Arabic and Kurdish.  Teams included both males and females conducted household survey/interviews.  Three questionnaires were administered: 1) general questions, 2) mental health, and 3) women’s health. | 115 clusters in Anbar, Baghdad, Nineveh, and Wasit not visited due to insecurity. | Questionnaires and forms sent to Federal Ministry of Health in Iraq for verification; incorrect or incomplete forms sent back to respective governorates.  Data double entered and verified. | Mortality and morbidity rates for pre- and post-invasion periods were calculated. The CMR pre-invasion was 3.17.  The VMR was calculated after accounting for sampling errors and adjusting for missing clusters. |

Table 2: Study Characteristics

Passive reporting – published studies

| **Author(s)** | **Study Type** | **Data Source** | **Methods** | **Included/Excluded** | **Validation Tools** |
| --- | --- | --- | --- | --- | --- |
| PDAR  2003 | Surveillance-based; mixed-methods.  *Analysis and synthesis of information from several sources* | Published – reports on secondary info source. | Analysis and synthesis of: journalistic surveys of casualty incidents, hospitals, burial societies, and graveyards, media and NGO reports of compilations of individual casualty incidents including and based on testimony from eyewitnesses, hospital personnel, aid workers, and the families of the dead. Combat deaths derived largely from embedded journalists and interviews with military personnel. | Includes deaths that occurred during the “war” (presumably up to official US declaration that combat had ceased, May 2003) | Determined by source information. |
| UNAMI  2006 | Facility-based; reported deaths.  *Synthesis of deaths reported by Ministry of Health and Medico-Legal Institute of Baghdad (M-LIB)* | Published – reports from primary info source. | Deaths reported by MOH derived from hospitals in all Governorates.  Deaths reported by M-LIB.  Reported deaths for each combined to produce total deaths. | MOH reports excluded Region of Kurdistan. | Confirmed deaths at hospitals and bodies arriving at M-LIB. |
| Brookings Institute  2008 | Surveillance- and facility-based; mixed-methods.  *Synthesis of variety of sources of information* | Published – reports from secondary info source. | Combatant death data comes from Iraq Minister of Interior, military personnel and media reports.  Non-combatant death data combines IBC as base data and increases by a factor of 1.75 (to account for the differences between the IBC and Ministry of the Interior figures) then combines with deaths estimated from crime rate analysis; the later period is based on UNAMI reports.  For the period from January to December 2007, based on U.S. State Department Weekly Status Report, September 12, 2007 and press briefings. | Civilian and military/police deaths reported separately; police and defense forces removed from civilian death totals.  Total deaths not provided; have not been added together given different time periods. | Not able to determine.  Based on rigor of source reports compiled. |
| Iraq Body Count  Ongoing  (info collected January 2008) | Surveillance-based; mixed-methods.  *Synthesis of media reports and eyewitness accounts* | Published – reports from secondary info source. | Survey of online (English language sites) newsgathering agency reports and eyewitness accounts.  Ranges given for discordant reports.  Reporting agency must meet base standards outlined.  Numbers must be independently reported by 2 or more agencies. | Includes only civilian deaths due to violence resulting from coalition military intervention, including insurgent and terrorist attacks and increased crime rate that resulted. | Must be independently report by 2 or more agencies.  Relies on rigor of reporting agency.  Reports are independently checked by 2 IBC staff and original compiler. |
| Just Foreign Policy  2008 | Survey- and surveillance-based; mixed-methods.  *Combination of Burnham et al. study results and IBC* | Published – based on primary research report plus secondary info source. | Combines the results of the Burnham et al. study with ongoing totals from the Iraq Body Count.  Formula is:  [Burnham et. al estimate as of July 2006] x [ (Current IBC Deaths) / (IBC Deaths as of July 1, 2006) ] | Includes civilian deaths due to violence. | Only as done through base studies and reports. |

Table 3: Study Characteristics

Passive reporting – unpublished studies

| **Author(s)** | **Study Type** | **Data Source** | **Methods** | **Included/Excluded** | **Validation Tools** |
| --- | --- | --- | --- | --- | --- |
| People’s Kifah  2003 | Survey- and facility-based; mixed-methods.  *Compilation of reports, interviews and personal accounts* | Unpublished – media reporting on secondary info source. | Volunteer based nation-wide survey gathering information from villages, towns and cities.  Hospital information.  Interviews with grave-diggers.  Eye-witness accounts. | Includes civilian deaths only.  Excluded Kurdish areas. | Not able to determine. |
| Iraqiyun  2005 | Survey- and surveillance- based; mixed-methods | Unpublished – media reporting on secondary info source. | Information gathered from relatives.  Information gathered from hospitals across the country. | Only violent deaths included.  Only deaths where relatives have been informed, not including abducted, assassinated or disappeared. | Not able to determine. |
| Iraq Ministry of Health  2006 | Facility-based; reported deaths in Baghdad area.  *Death rates reported to Ministry; data extrapolation since March 2003 for total mortality figures* | Unpublished – media reporting on primary info source. | 75-100 deaths per day reported at morgues and hospitals in Baghdad.  Current daily death rate extrapolated to determine total death estimate since 2003. | All deaths due to violence. | Not able to determine.  Based on count of dead bodies.  Confirmed by Ministry staff. |
