# Supplementary material for: Iraq War mortality estimates: A systematic review
Source: Confl Health. 2008 Mar 7;2:1. doi: 10.1186/1752-1505-2-1 (PMC2322964; doi:10.1186/1752-1505-2-1)
Supplement: Additional file 2 — Table 4: Study Results. This chart presents the detailed study outcomes, including mortality totals and rates. [file 1752-1505-2-1-S2.doc]

Table 4 – Study Results

| **Author(s)** | **Date of Data Collection** | **Period of data collection** | **Excess deaths due to all causes since start of invasion**  **[95% CI] (range)*** | **Combatant Mortality** | **Non-combatant Mortality** | **Deaths attributable to Violence** | **Deaths Per Day** | **Crude and/or Violent Mortality Rate**  **(per 1,000)** |
| --- | --- | --- | --- | --- | --- | --- | --- | --- |
| POPULATION-BASED STUDIES | |  |  |  |  |  |  |  |
| Roberts  2004 | Sept. 2004 | March (20), 2003 to  Sept. (15) 2004  (approx. 545 days) | 98,000  [8,000 to 194,000] | N/A | N/A | 24%  (exc. Falluja)  51%  (inc. Falluja) | 180 deaths / day  (15 – 356) | 12.3 total CMR* |
| Iraq Living Conditions Survey  2004 | April – May, 2004 | March 20, 2003  to  May (31) 2004  (approx. 438 days) | 24,000  [18,000 to 29,000] | N/A | N/A | 24,000  (18,000 to 29,000) | 54 deaths / day  (41 – 66) | 0.74 VMR  (0.55 – 0.89) |
| Burnham  2006 | May - July, 2006 | March (20), 2003 to  July (15), 2006  (approx. 1213 days) | 654,965  [392,979 to 942,636] | N/A | N/A | 601,027  (426,369 to 793,663) | 540 deaths / day  (324 – 777) | 13.2 total CMR*  7.2 VMR*  (4.36 – 10.45)  7.26 Excess CMR**  (4.36 - 10.45) |
| Opinion Research Business  2007 | August 2007 | March (20), 2003 to  August (15), 2007  (approx. 1609 days) | N/A | N/A | N/A | 1,220,580  (733,158 to 1,446,063) | 759 deaths / day  (456 – 899) | 10.25 total VMR  (6.15 – 12.16) |
| Iraq Family Health Survey  2008 | 2006 and 2007 | March (20), 2003 to  June (30), 2006  (approx. 1198 days) | 433,000*** ^+^  ^[354,000 to 523,000]^ | N/A/ | N/A | 151,000  [104,000 to 223,000] | 126 deaths / day  (87 – 186) | 1.09 VMR*^+^  [0.81-1.50]  2.84 Excess CMR** |
| PASSIVE REPORTING *– Published Studies* | | | | | | | | |
| PDAR  2003 | Synthesis of results with different collection dates | March 19, 2003  to  May 1, 2003  assumed to be the duration of ‘the war’  (approx. 42 days) | N/A | 9,200  (+/- 1,600) | 3,750  (+/- 500) | 12,950  (+/- 2,150)  (not stated but inferred based on data collected) | Total:  308 deaths / day  Combatant:  219 deaths / day  Non-combatant:  89 deaths / day | Total:  4.15 VMR  Combatant:  2.95 VMR  Non-combatant:  1.2 VMR |
| UNAMI  2006 | Monthly  May – Dec., 2006 | May 1, 2006  to  December 31, 2006  (approx. 245 days) | N/A | N/A | 25,847 | 25,847 | 106 deaths / day | 1.42 VMR |
| Brookings Institution  2007 | Synthesis of results with different collection dates | Combatant:  June (1), 2003 to January 16, 2008  (approx. 1661 days)  Non-combatant:  May (1), 2003 to Dec. 31, 2007  (approx. 1706 days) | N/A | 7,792  (includes military and police) | 97,017 | All  (civilian deaths all due to violence; though not stated, the assumption is that military and police deaths also due to violence) | Combatant:  5 deaths / day  Non-combatant:  57 deaths / day | Combatant:  0.06 VMR  Non-combatant:  0.77 VMR |
| Iraq Body Count  Ongoing  (info collected January 17, 2008) | Daily  Ongoing | March 20, 2003 to  January 17, 2008  (approx. 1764 days) | N/A | N/A | 84,333  (80,621 to 88,044) | 84,333  (80,621 to 88,044) | 48 deaths / day  (46 - 50) | 0.64 VMR  (0.61 - 0.67) |
| Just Foreign Policy  Ongoing  (info collected January 17, 2008) | Daily  Ongoing | March 20, 2003 to  January 17, 2008  (approx.1764) | N/A | N/A | 1,168,058 | 1,168,058 | 662 deaths / day | 8.95 VMR |
| PASSIVE REPORTING *– Unpublished Studies* | | | | | | | | |
| People’s Kifah  2003 | Sept - Oct., 2003 | March 20, 2003  to  Oct. (15) 2003  (approx. 209 days) | N/A | N/A | 37,137 | 37,137 | 177 deaths / day | 2.39 VMR |
| Iraqiyun  2005 | Unknown | March 20, 2003  to  July 11, 2005  (approx. 844 days) | N/A | N/A | N/A | 128,000 (uncertain) | 152 deaths / day | 2.03 VMR |
| Iraq Ministry of Health  2006 | Average monthly death rates.  Ministry began keeping records in early 2004. | March (20), 2003 to  Nov. (30) 2006  (approx. 1351 days) | N/A | N/A | N/A | 100,000  to  150,000 | 75-100 deaths / day | 1.01 – 1.34 VMR |

* As reported by the study authors

** Excess mortality estimates include all deaths, both direct and indirect in excess of the pre-invasion baseline.

*** Calculated as: 151,000 x 2.84 / 0.99 = 433,171 (Excess VMR calculated as 1.09 - 0.10 = 0.99)

^+^ Based on rates provided in Table 3 in manuscript

N/A: Not applicable or unreported
